# Diseases of the Army at Monterey

**Published:** 1847-06

**Authors:** N. S. Jarvis

**Affiliations:** Surgeon U. S. A.


*Diseases of the Army at Monterey.—By* Dr. N. S. Jarvis, *Surgeon U. S. A.*—The first wounds were received in crossing the plain, and were inflicted by grape and cannon-shot. This was of course before we had approached within reach of their musketry. These wounds were all low; generally at or just above the ankle, according to distance and direction. Of the first three men brought to me, two had received wounds from twelve pound shots just above the ankle, which had nearly severed the limbs, which were hanging only by a portion of integuments. The other had his heel torn off by a six pound shot. Shortly after, our troops having advanced within reach, and under the fire of the Mexican infantry, numerous cases of wounds by musket and escopette* balls were brought to me; these latter are one-third larger than our musket balls, and consequently inflict a more severe and formidable wound. So numerous at this time became the wounded in our pit, and so constant and heavy the fire directed towards the parties approaching with the wounded, as to compel us to remove our hospital several hundred yards farther in the rear. We had not long been in our new position, when some covered wagons bringing the wounded attracted the attention of the enemy, who immediately re-opened their fire, compelling us a second time to remove beyond the range of their shot.

Among the numerous projectiles, occasioning severe and fatal wounds, were grape, canister, fragments of iron and copper shells, and stones knocked by the balls from the buildings and walls. Their shells were thrown with great accuracy, frequently in the midst of a body of troops, but fortunately killing and wounding but few.

Before speaking of any particular wounds, I will here take occasion to make some remarks respecting the character they assumed, and the peculiar causes acting to prevent a favorable result, so far as regarded the healing of all, even the most slight. The first annoyance we experienced, and which no doubt exerted an injurious effect, was one little anticipated at the time. The moment a limb was amputated numerous flies would alight on the stump, and must have deposited their eggs, for when it became necessary to dress the stump, myriads of maggots were found buried in it, which could be expelled with great difficulty—rendering it necessary, in some instances, to reopen the flap, for their complete extermination. A much more formidable enemy made its appearance in an erysipelatous inflammation of the integuments covering the stump, which generally set in two or three days after the operation; and, notwithstanding all the means made use of to arrest it, most commonly ended in sloughing, and either proved fatal or rendered a second amputation necessary. That some influence existed previously, either external or internal, from causes connected with the state of the atmosphere, or habits

---

* An escopette is a short carbine, similar to a blunder-buss, and carries a ball one-third larger than our musket.—M.

of the men, arising from diet or water, was manifest. The slightest wound or scratch became in every case a tedious ulcer, in some instances proving a cause for serious alarm. Apparently the most trifling wounds required an unusual time for healing, and even those that had previously healed would break out again, and present greater difficulty in their cure than in the first instance.

At this period no atmospheric causes apparently existed to produce this unfavorable aspect of things. Nothing could exceed the loveliness of the weather, if I may so express myself, and if the middle of the day were warm, the morning and evening refreshed us by a most delightful temperature and cloudless sky. No rain had fallen, with the exception of one or two showers, for nearly a month, and consequently little moisture existed to produce its well-known morbific influence. Immediately after the capitulation of the city, on the 25th of September, all the wounded of the different divisions entered the town, and suitable buildings were provided for their accommodation. Upwards of two hundred officers and men from the 1st and 3d divisions, who had been most severely wounded, were coveyed thither on the same day in litters and wagons. The wounded of the 2d division already occupied the city.

Our camp afforded no comfort nor shelter for them beyond a few small tents and a solitary blanket laid on the ground; and many were destitute of even this apology for a bed, having lost them on our march. Many had no other clothing than that in wear, which was not only torn and soiled in climbing over the hedges, walls, &c., during the battle, but was stiff and saturated with blood from their wounds. A few days after their reception into the hospitals, tertian intermittent fever made its appearance, attacking many of the wounded, and, in the majority, retarding or completely arresting convalescence. On many of those severely wounded it exerted a decidedly pernicious influence, and no doubt contributed, in some cases, to a fatal termination. It not only attacked the wounded in the hospitals, but prevailed extensively in camp and among the population of the town and neighboring country. I cannot say to what extent this may be attributed to the putrid exhalations arising from the numerous bodies of men and horses slain in the different combats, and which had been slightly covered with earth, and emitted a most sickening and offensive effluvia. This, doubtless, contributed largely towards infecting or destroying the purity of the air, and establishing a poisonous miasm.—*New York Jour. of Medicine.*

